# Supplementary material for: Association between oral microbiome and seven types of cancers in East Asian population: a two-sample Mendelian randomization analysis
Source: Front Mol Biosci. 2023 Nov 21;10:1327893. doi: 10.3389/fmolb.2023.1327893 (PMC10702768; doi:10.3389/fmolb.2023.1327893)
Supplement: Supplementary file 1 [file DataSheet1.ZIP › Supplementary Materials/MR plots for tongue/tongue═╝/Colorectal cancer/pheno.674_to_colorectal cancer_forest.pdf]

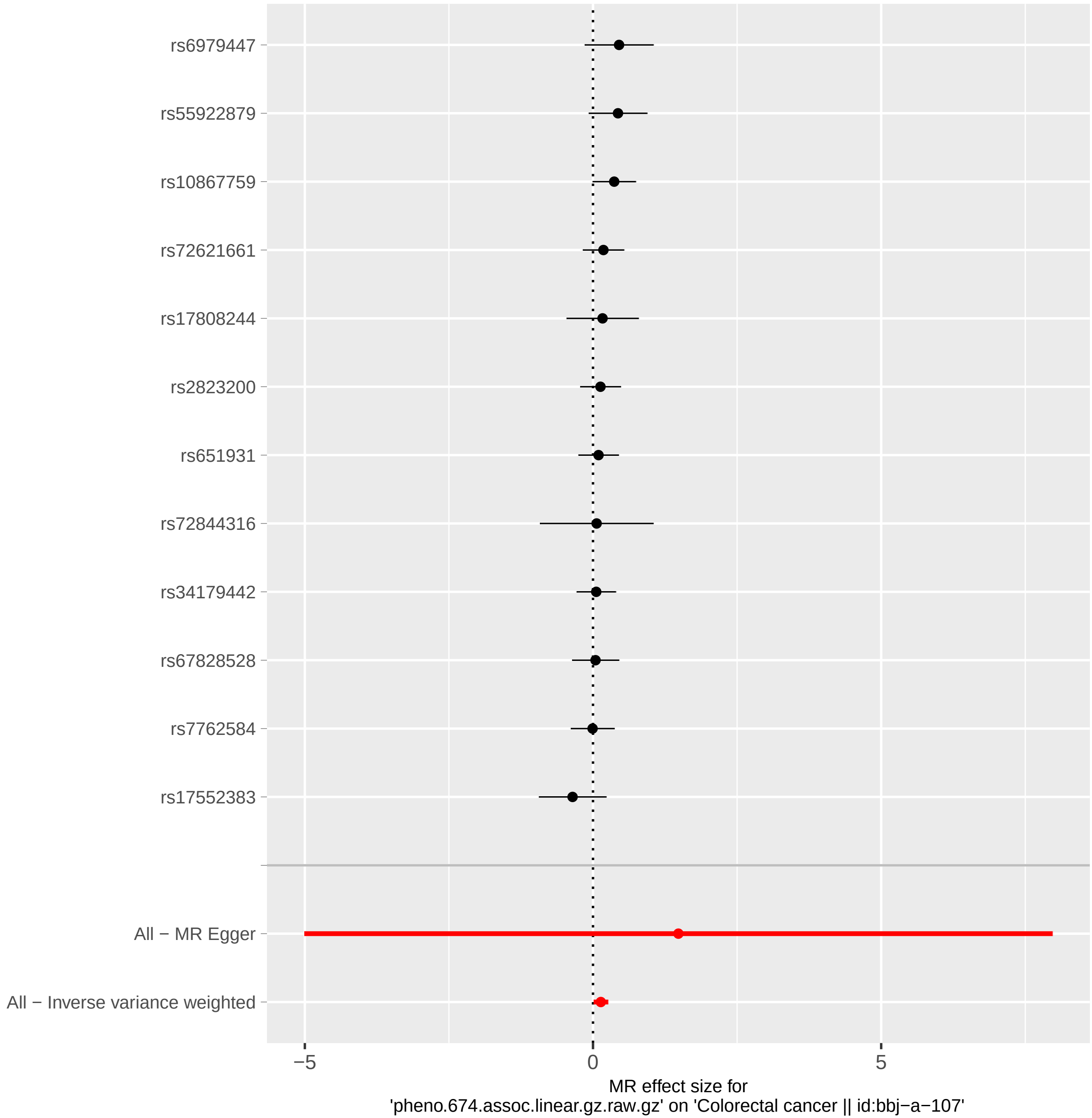

rs6979447
rs55922879
rs10867759
rs72621661
rs17808244
rs2823200
rs651931
rs72844316
rs34179442
rs67828528
rs7762584
rs17552383
All − MR Egger
All − Inverse variance weighted
−5
0
5
MR effect size for
'pheno.674.assoc.linear.gz.raw.gz' on 'Colorectal cancer || id:bbj−a−107'
